# Eleutherin and Isoeleutherin Activity against *Staphylococcus aureus* and *Escherichia coli* Strain’s: Molecular Docking and Antibacterial Evaluation

**DOI:** 10.3390/ijms252312583

**Published:** 2024-11-23

**Authors:** Mírian Letícia Carmo Bastos, Houéfa Egidia Fallon Adido, Ananda Karolyne Martins de Brito, Cristian Kallahan Silva Chagas, Ana Laura Gadelha Castro, Gleison Gonçalves Ferreira, Pedro Henrique Costa Nascimento, Walice Rans da Silva Padilha, Rosana Moura Sarmento, Viviane Vasconcelos Garcia, Andrey Moacir do Rosario Marinho, Patrícia Santana Barbosa Marinho, Johnatt Allan Rocha de Oliveira, Valdicley Vieira Vale, Sandro Percário, Maria Fâni Dolabela

**Affiliations:** 1 Biodiversity and Biotechnology Bionorte Network, Federal University of Para, Belém 66075-110, PA, Brazil; mirian.c.bastos@hotmail.com (M.L.C.B.); percario@ufpa.br (S.P.); 2 Faculty of Pharmacy, Federal University of Para, Belém 66075-110, PA, Brazil; adidofallon@gmail.com (H.E.F.A.); pedrohcn@gmail.com (P.H.C.N.); walicerans@gmail.com (W.R.d.S.P.); 3 Postgraduate Program in Pharmaceutical Sciences, Federal University of Para, Belém 66075-110, PA, Brazil; anandakarolyne14@gmail.com (A.K.M.d.B.); criskallahan@gmail.com (C.K.S.C.); gleisonhist@gmail.com (G.G.F.); 4 Postgraduate Program in Pharmaceutical Innovation, Federal University of Para, Belém 66075-110, PA, Brazil; lauracastro.farmacia3@gmail.com (A.L.G.C.); valdicleyvale@gmail.com (V.V.V.); 5 Faculty of Pharmacy, University of the Amazon—UNAMA, Belém 67113-901, PA, Brazil; rosana.sarmento@hotmail.com (R.M.S.); vivianev.garcia@yahoo.com.br (V.V.G.); 6 Postgraduate Program in Chemistry, Federal University of South and Southeast Pará, Marabá 68380-000, PA, Brazil; andrey@ufpa.br; 7 Postgraduate Program in Chemistry, Federal University of Pará, Belém 66075-110, PA, Brazil; psbmarinho@yahoo.com.br; 8 Postgraduate Program in Food Science and Technology, Federal University of Pará, Belém 66075-110, PA, Brazil; johnatt@ufpa.br

**Keywords:** antibacterial activity, *Eleutherine plicata*, molecular docking, naphthoquinones

## Abstract

Naphthoquinones eleutherin and isoeleutherin have demonstrated promising antibacterial activity, probably due to their quinone structure, which can generate reactive oxygen species. The study examines the activities of pathogens, such as *Staphylococcus aureus* and *Escherichia coli*, associated with antimicrobial resistance and explores their potential mechanisms of action. The MIC, IC_50_, and MBC were determined. PharmMapper 2017 server and GOLD 2020.1 software were utilized for molecular docking to identify protein targets and interaction mechanisms. The docking predictions were verified by redocking, focusing on structures with RMSD below 2 Å. The molecular docking revealed a significant affinity of eleutherin for the peptide, transcriptional regulator QacR, and regulatory protein BlaR1 with better interactions with BlaR1 than the crystallographic ligand (benzylpenicillin). Isoeleutherin demonstrated specific interactions with methionine aminopeptidase, indicating specificity and affinity. In summary, the difference in naphthoquinones activities may be related to structural differences. Eleutherin exhibits potential as a therapeutic adjuvant to reverse bacterial resistance in *S. aureus*, suggesting this molecule interferes with the antibiotic resistance mechanism. The absence of homologous proteins or variations in the structure of the target proteins could be the cause of the inactivity against *E. coli*.

## 1. Introduction

Traditional plant knowledge is crucial for the discovery of bioactive compounds and serves as a strong basis for scientific research. A notable example is *Eleutherine bulbosa* (Mill.) Urb., which has as a synonym *Eleutherine plicata* Herb. and is a native American plant that occurs in several tropical countries [1,2]. It is widely used in traditional Amazonian medicine to treat malaria, gastric ulcers, intestinal disorders, amoeba and other parasitic infections, dysentery and diarrhea from bacterial origin, hemorrhoids, and menstrual disorders [3,4,5].

Several compounds in this plant were identified through phytochemical analyses, including quinones and terpenes. Chemical studies led to the isolation of isoeleutherin; eleutherin; eleutherol; eleutherinone; (R) 4-hydroxyeleutherin; eleuthone; isoeleuthoside C; and eleutherinol-8-O-b-D-glucoside (Figure S1) [6,7,8].

Other studies evaluated the potential binding targets of eleutherin and isoeleutherin. Molecular docking studies for malaria showed that the process is similar to that of atovaquone. The interactions with conserved residues in the binding cavity of the cytochrome bc1 complex are involved. This protein is found in the parasite’s mitochondria. Antioxidant defense enzymes may play a role in regulating oxidative stress through interaction [9]. In silico studies showed that these naphthoquinones can stabilize the topoisomerase II complex [10] and act in the apoptosis pathway [11], inhibiting enzymes involved in the biosynthesis of nucleic acids in the energy metabolism of *Plasmodium falciparum*, in addition to inducing oxidative stress [4].

The activity of *E. plicata* against bacteria has also been evaluated. One study demonstrated the plant’s efficacy against Staphylococcus, with the dichloromethane fraction exhibiting the most notable activity [2]. This activity has been related to isoeleutherin and eleutherin. However, the targets involved are probably different. Among the targets of *S. aureus* are the metalloenzyme peptide deformylase (PDF), the transcriptional regulator QacR, the sensory regulatory protein BlaR1, and the monomeric methionine aminopeptidase (MetAP).

PDF acts in the ribosome translation process [12] encoded by the def gene, essential for both bacterial growth and survival [12,13], catalyzing the removal of the formyl group from the N-terminal methionine residue of newly synthesized polypeptides, necessary for proper protein folding and function [14]. The regulator QacR is a repressor protein responsible for controlling the expression of the multidrug efflux pump QacA in *S. aureus*, contributing to bacterial resistance [15].

BlaR1 has been demonstrated to detect β-lactam antibiotics and subsequently transmit this information to the cytoplasm. In the MRSA strain, resistance to β-lactam antibiotics is mediated by this protein [16]. MetAP is a binuclear metalloprotease that is essential for cell growth in organisms such as *S. aureus*, which contains cobalt and exhibits catalytic properties in removing the N-terminal methionine from newly synthesized proteins [17].

*Staphylococcus aureus*, particularly methicillin-resistant *S. aureus* (MRSA), is a significant contributor to hospital- and community-acquired infections, leading to treatment failures and increased mortality [18]. *E. coli*, specifically ESBL-producing strains, has shown resistance to various antibiotics, making treatment of urinary tract infections and sepsis more challenging [19]. ESBL-resistant *Escherichia coli* strains are frequently responsible for urinary tract infections and are associated with high rates of therapeutic failure due to their resistance to commonly used antibiotics [20].

Thus, the present study investigated the activity of eleutherin and isoeleutherin against *S. aureus* and *E. coli* and the intermolecular interactions between naphthoquinones and protein targets involved in the action. It is expected that these results will highlight this plant’s therapeutic potential and may contribute to the development of new antimicrobial agents based on natural compounds.

## 2. Results

### 2.1. Chemical Studies

The EE (yield = 10% in relation to the dry material) and its hexane (19.2%), dichloromethane (38.5%), ethyl acetate (19.6%), and methanolic (22.6%) fractions were subjected to phytochemical analysis. Bands suggestive of naphthoquinones were observed in all fractions. From the FrDcm, 35 sub-fractions were obtained and analyzed in TLC and grouped by similarity of the bands.

The sub-fraction Fr 22–23 presented a band with Rf equivalent to that of eleutherin, and after recrystallization, yellow/orange crystals were obtained. Analysis of fraction 27 indicated the presence of a single band with the same Rf as isoeleutherin, which became purer after recrystallization. The Fr 22–23 and Fr 27 sub-fractions (after recrystallization) were subjected to 1H NMR analyses. The 1H NMR results demonstrated sub-fraction Fr 22–23 was eleutherin (Figure S6) and Fr 27 was its isomer and isoeleutherin (Figure S7).

### 2.2. Antibacterial Activity of Eleutherin and Isoeleutherin

In evaluating the eleutherin and isoeleutherin activity against Gram-positive (*S. aureus*) and Gram-negative (*E. coli*) bacteria, a relationship between bacterial inhibition and the concentration of the compounds was noted. The results indicated a greater inhibitory potential in *S. aureus* when compared to *E. coli* (Table 1).

**Figure 1:**
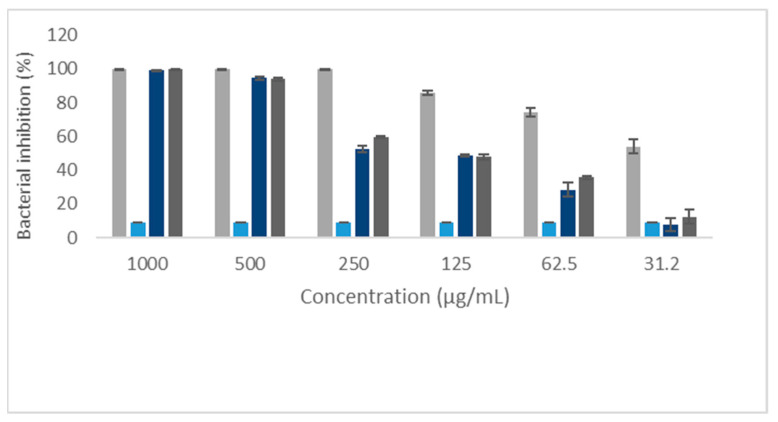
Bacterial inhibition of *Staphylococcus aureus* by eleutherin, isoeleutherin, and controls. Solvent—methanol.

**Figure 2:**
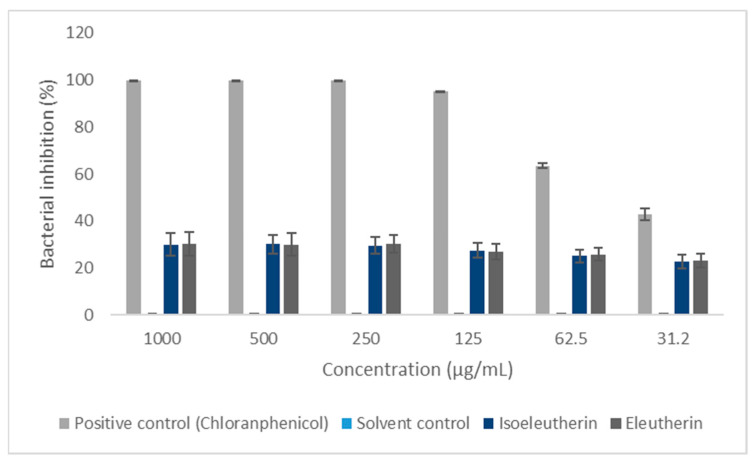
Bacterial inhibition of *Escherichia coli* by eleutherin, isoeleutherin, and controls. Solvent—methanol.

Furthermore, it was observed that the solvent did not interfere with bacterial growth in *S. aureus* and *E. coli* strains (Figure 1 and Figure 2).

Isoeleutherin and eleutherin were found to be inactive against *S. aureus* and *E. coli* when the MIC was determined (Figures S2–S5). A similar fact was observed for the MBC; however, when the IC_50_ was determined, the naphthoquinones were moderately active against *S. aureus* (Table 2).

### 2.3. Target Proteins Involved in the Eleutherin and Isoeleutherin Action

To determine the targets of action involved in the activity against *S. aureus*, a preliminary study was performed using the PharmMapper 2017 program. In the case of eleutherin, the activity may be related to three different proteins, while for isoeleutherin, the results suggest the involvement of one protein (Table 3).

### 2.4. Molecular Docking of Eleutherin and Isoeleutherin in S. aureus Proteins

The crystallographic ligands (actinonin and pentamidine) in the case of PDF and QacR regulator showed higher CS and GS values compared to eleutherin. Likewise, all these ligands demonstrated higher Van der Walls energy compared to naphthoquinones (Table 4). BlaR1 shows a distinct behavior, with eleutherin displaying similar CS, GS, Van der Walls energy, and ΔG values to benzylpenicillin, indicating a comparable binding profile between the two molecules and the target.

In the interactions with MetAp, when comparing isoeleutherin and ketoheterocycle 618, a similar binding profile with close values of CS, GS, and ΔG was observed. The Van der Walls energy was higher for isoeleutherin (Table 4).

**Figure 3:**
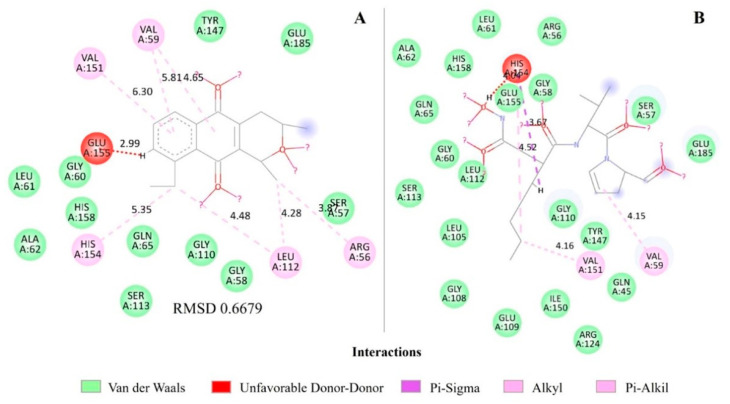
Molecular interactions of eleutherin and actinonin with peptide deformylase–PDF. (**A**) Interactions of eleutherin; (**B**) interactions with the crystallographic ligand actinonin; and RMSD—Root Mean Square Deviation, value in angstrom.

When analyzing the molecular interactions for PDF, both eleutherin and actinonin show similar binding patterns in the active site of the protein (Figure 3). Both present Van der Waals interactions with similar residues, such as TYR A:147, GLU A:185, SER A:57, LEU A:61, GLN A:65, GLY A:110, and GLY A:58, with similar distances (Table 4 and Table S1; Figure 3), suggesting they comparably occupy the binding site.

The two ligands present an unfavorable interaction (eleutherin with GLU A: 155 at 2.99 Å and actinonin with HIS A: 154 at 2.67 Å), indicating a possible steric conflict or repulsion. Based on the analysis of Pi-Alkyl and Alkyl interactions, eleutherin and actinonin show a balanced distribution of these interactions. Actinonin presents two Alkyl interactions (VAL 59 and VAL 151) and one Pi-Sigma interaction (HIS 154). Eleutherin has two Pi-Alkyl interactions (VAL 59 and VAL 151) and Alkyl interactions (LEU 112, ARG 56, HIS 154, VAL 59; Figure 3; Table S1).

**Figure 4:**
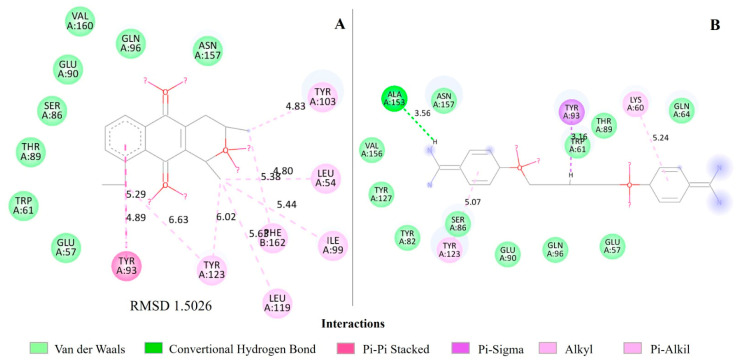
Molecular interactions of eleutherin and pentamidine with the regulator QacR. (**A**) Interactions of eleutherin; (**B**) interactions with the crystallographic ligand pentamidine; and RMSD—Root Mean Square Deviation, value in angstrom.

For the QacR regulator, eleutherin forms significant interactions with specific residues in the binding site, with a predominance of Pi-Pi stacked and Alkyl interactions. Residues such as TYR 93 and TYR 123 show both Pi-Pi stacked and Alkyl interactions, indicating a strong and diverse binding with eleutherin, and the distances range from approximately 4.80 Å to 6.63 Å. Pentamidine mainly forms Pi-Sigma, Alkyl, and conventional hydrogen bond interactions with residues in the protein 2 binding site, with distances ranging from approximately 3.16 Å to 5.24 Å, with emphasis on the formation of a conventional hydrogen bond with LYS 60 (Figure 4; Table S1).

**Figure 5:**
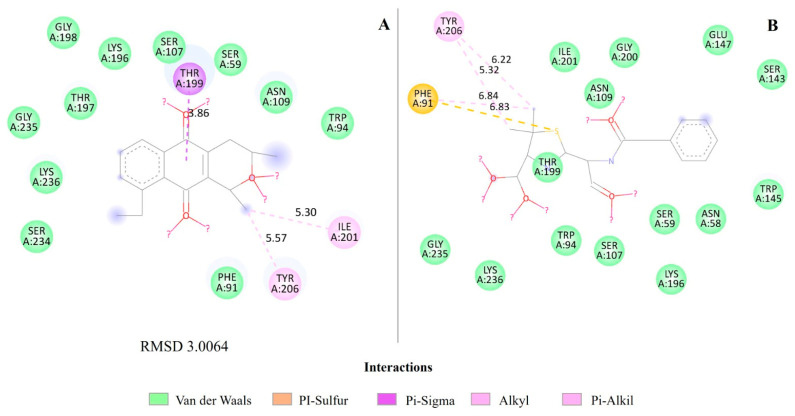
Molecular interactions of eleutherin and benzylpenicillin with the transcriptional regulator BlaR1. (**A**) Interactions of eleutherin; (**B**) interactions with the crystallographic ligand benzylpenicillin; and RMSD—Root Mean Square Deviation, value in angstrom.

Related to BlaR1, eleutherin apparently forms specific favorable interactions (Pi-Sigma and Alkyl with shorter distance) that may be stronger and better accommodated by the binding site of this target—a Pi-Sigma bond at residue TYR 199, indicating a planar interaction between the Pi group of eleutherin and the Sigma system of the tyrosine residue, Alkyl bonds with residues ILE 201 and TYR 206. Benzylpenicillin interacted as Pi-sulfur with PHE 91 and varied distances of Van der Waals interactions with other residues (Figure 5; Table S1).

**Figure 6:**
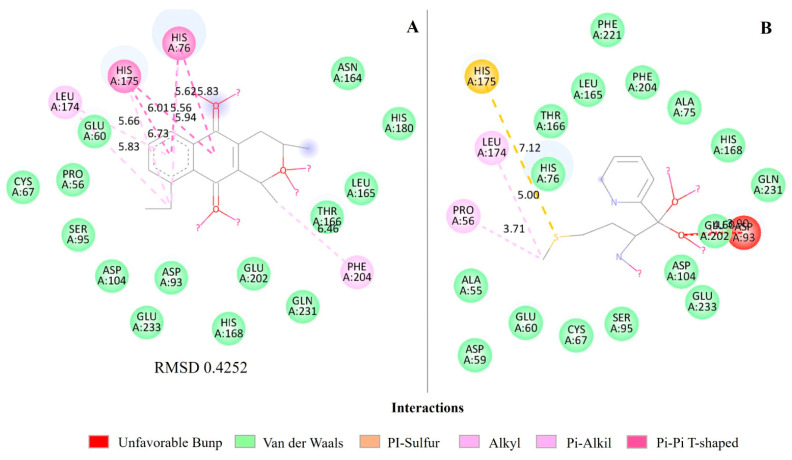
Molecular interactions of isoeleutherin and ketoheterocycle 618 with methionine aminopeptidase—MetAP. (**A**) Interactions of isoeleutherin; (**B**) interactions with the crystallographic ligand ketoheterocycle 618; and RMSD—Root Mean Square Deviation, value in angstrom.

In the intermolecular interactions of MetAP, isoeleutherin exhibits a variety of more specific interactions, such as Pi-Pi T-shaped and Pi-Alkyl, while ketoheterocycle 618 exhibits mainly Van der Waals and Pi-sulfur interactions. For HIS 76, for example, isoeleutherin exhibits Pi-Pi T-shaped interactions in two different conformations and one Alkyl interaction. Ketoheterocycle 618 interacts with HIS 76 mainly through Van der Waals bonds, indicating a less specific and more distant interaction compared to isoeleutherin (Figure 6; Table S1).

HIS 175 also exhibits T-shaped Pi-Pi interactions in two different conformations, in addition to an Alkyl interaction with isoeleutherin; meanwhile, with ketoheterocycle 618, it forms a specific Pi-sulfur interaction of 7.12 Å. LEU 174 interacts mainly through Alkyl and Pi-Alkyl bonds with isoeleutherin, around 5.66 Å and 5.83 Å, indicating close contact, but also interacts through an Alkyl bond with ketoheterocycle 618 at 5.00 Å. PHE 204 forms an Alkyl interaction with isoeleutherin at a distance of 6.46 Å, which does not occur with ketoheterocycle 618, which interacts through Van der Walls bonds (Figure 4).

The RMSDs of the PDF with actinonin interactions (Figure 3), QacR regulator with pentamidine (Figure 4), and MetAP with ketoheterocycle 618 (Figure 6) were less than 2 Å. However, the redocking of BlaR1 with benzylpenicillin had an RMSD greater than 2 Å (Figure 5).

## 3. Discussion

Eleutherin and isoeleutherin are promising molecules in terms of biological activities [2,4,10]. The structure of eleutherin and isoeleutherin differs in the presence of a single chiral center in the pyran ring, along with the functionality of the methyl group. Isoeleutherin has a pyran ring with a methyl group in a specific configuration, while eleutherin has a slightly different structure [21]. These structural changes may be involved in the differences between the targets in *S. aureus*, inhibited by eleutherin but not inhibited by isoeleutherin, resulting in different therapeutic potential.

In this study, we investigated their activities against *S. aureus* and *E. coli* and explored the possible mechanisms of action. Both naphthoquinones inhibited *S. aureus* (Table 2) activity with eleutherin showing more. When comparing these results with other antimicrobial activity studies carried out with naphthoquinones, a synergistic effect was observed, preventing the development of resistant strains of *S. aureus* when associated with another antimicrobial [22]. Other studies corroborate these findings, demonstrating the efficacy of extracts and fractions of *E. plicata* against *S. aureus* [2,23,24] and the absence of activity against *E. coli* [25].

A study demonstrated, through agar diffusion and microdilution tests against *S. aureus*, that the dichloromethane fraction (DF) was the most active with a minimum inhibitory concentration (MIC) of 125 μg/mL, indicating strong inhibitory activity. The fraction containing isoeleutherin, obtained from DF, had an MIC of 250 μg/mL. For all samples, the minimum bactericidal concentration (MBC) was greater than 1000 μg/mL, suggesting that the substances present in this plant were not able to eliminate the bacteria, only inhibit them [26].

The findings of our study indicate the eleutherin activity does not involve a bactericidal effect. Several bacterial signaling pathways may be involved in this effect, and it is necessary to understand how naphthoquinones and crystallographic ligands act on *S. aureus* targets. Eleutherin, which had the greatest antimicrobial activity, interacted with different proteins in *S. aureus*: PDF, regulator QacR, and BlaR1, unlike isoeleutherin, which interacted with a single target, MetAP (Table 3).

PDF is involved in the bacterial translation process, and eleutherin, when binding to this protein, it interferes with this process and can lead to an accumulation of defective proteins that are functional. PDF inhibitors, such as actinonin, can be bactericidal, as they interfere with essential protein synthesis, leading to cell death [27]. When comparing eleutherin with actinonin, they had almost compatible stability in the docking study (Table 4; Figure 3). Regarding the distances, they are reasonably close. However, actinonin presents some slightly smaller distances, which indicates some advantage in binding efficiency. Eleutherin and the control showed the same binding pattern in the active site (Figure 3; Table S1). These interactions are important for the stabilization of the protein–ligand complex (Table 4).

Since actinonin is bactericidal, the same was expected for eleutherin, but it was not observed. The bacteriostatic activity observed in the present study may be related to the characteristics of the *S. aureus* ATCC 6538 strain, which is considered moderately resistant to penicillin. Perhaps the result would be bactericidal if the strain was sensitive to penicillin.

Eleutherin forms more specific and stronger interactions (Figure 4 and Figure 5), such as Pi-Pi stacked with QacR, which are considered robust and specific, and the distances suggest a good proximity for specific and stable interactions with amino acid residues in the binding site of the QacR regulator (Figure 4; Table S1). The QacR regulator is involved in bacterial multidrug resistance because it regulates the expression of the QacA efflux pump. Inhibitors of this protein, such as pentamidine, increase the susceptibility of bacteria to antimicrobials by preventing the expression of the efflux pump [28]. The binding free energy of eleutherin with the QacR regulator also indicates a spontaneous and thermodynamically favorable interaction (Table 4) [29], suggesting that eleutherin can effectively bind to this protein, inhibiting its regulatory function in the expression of the QacA efflux pump. In this context, eleutherin may be an important therapeutic adjuvant, reversing bacterial resistance to different classes of drugs.

BlaR1, another target of eleutherin, is a sensor part of a system that regulates resistance to β-lactam antibiotics in *S. aureus*. It controls the expression of β-lactamases and penicillin-binding proteins, such as PBP2a [30]. In the docking, eleutherin formed more specific and stronger bonds with BlaR1 compared to benzylpenicillin and also demonstrated a good ability to interact with the hydrophobic surface of the target through Van der Walls forces (Figure 5; Table S1), contributing to the total free energy [31], significantly favoring stability and high complementarity of the complex’s binding surface [32], and suggesting greater efficacy of eleutherin in preventing β-lactamase production (Table 2).

The binding of eleutherin to BlaR1 is an interaction that releases energy (negative enthalpy), corroborated by the highest ΔG values for this substance (Table 4). However, the Root Mean Square Deviation (RMSD) between the conformation of eleutherin and BlaR1 (Figure 5) was 3.0064 Å, indicating the structure departs from the conformation of the reference, the crystallographic ligand; that is, although eleutherin can bind to the target, there is significant conformational variability in the binding position over time. During binding, eleutherin may be inducing conformational changes in BlaR1 [33,34].

Inhibition of BlaR1 phosphorylation by synthetic kinase inhibitors reversed the resistance phenotype, restoring bacteria’s susceptibility to β-lactam antibiotics [35]. This inhibition may be related to the eleutherin effect, which may be concentration-dependent; thus, the use of higher concentrations of eleutherin can achieve greater effective inhibition of BlaR1 [36].

Isoeleutherin has been demonstrated to inhibit MetAP, thereby slowing down bacterial growth, a characteristic of bacteria-stopping agents. The interference in protein maturation caused by MetAP inhibition prevents newly synthesized proteins from reaching their correct functional conformation, leading to an accumulation of misfolded and dysfunctional proteins. This accumulation can interrupt essential cellular processes and slow bacterial growth, but does not cause immediate cell lysis [37]. This mechanism explains the possible bacteriostatic effect on *S. aureus* produced by isoeleutherin.

In molecular docking, MetAP and isoeleutherin showed comparable inhibition to the control, and both interactions are spontaneous and thermodynamically favorable (Table 4), with slightly better stability for ketoheterocycle 618, reflected in a more negative ΔG value (Table 4). Isoeleutherin presents more specific interactions with MetAP, and the crystallographic ligand ketoheterocycle 618 exhibits Van der Walls and Pi-sulfur bonds. The distances between MetAP residues and the ligands vary significantly between the two compounds (Figure 6; Table S1). However, isoeleutherin tends to be closer to the residues HIS 76, HIS 175, LEU 174, and PHE 204, indicating possible stronger and more specific interactions with this residue (Figure 6; Table S1).

In further analysis, the inactivity of eleutherin against *E. coli* could be explained by the absence of homologous proteins or structural differences in the same protein targets in these bacteria. For example, PDF in *E. coli* has structural variations [38] that reduce the binding affinity of eleutherin, while proteins such as QacR and BlaR1 do not have direct functional homologs in *E. coli* [39,40]. The same occurs with isoeleutherin, since MetAP in *E. coli* has structural variations that may reduce the binding affinity of isoeleutherin. The structure of *E. coli* MetAP reveals a novel structure and a cobalt-dependent active site, making it a new class of proteolytic enzyme [41,42].

Furthermore, the composition and organization of the cell wall in Gram-negative bacteria, such as *E. coli*, are more complex due to a peptidoglycan layer between the inner plasma membrane and the outer membrane. The presence of the outer membrane, with its porin proteins and active transport systems, provides an additional barrier, hindering the entry of many antibiotics and antimicrobial compounds [43,44]. These structural differences are crucial to understanding the variation in susceptibility of different classes of bacteria to antimicrobial agents [45], as observed in biological assays with eleutherin and isoeleutherin.

## 4. Materials and Methods

### 4.1. Chemical Studies

The bulbs of *E. plicata* were collected in Tracuateua, Pará, Brazil (lat. 1.1436°, long. 46.9551°), in July, and an exsiccate was deposited in the Museu Paraense Emilio Goeldi (MG 202631). The powder from the bulbs was subjected to exhaustive maceration with 96% ethyl alcohol for 21 days, with filtration of the extractive solution every 7 days and subsequent addition of fresh solvent. The extractive solutions were concentrated in a rotary evaporator under reduced pressure, obtaining the ethanolic extract (EE) of the bulbs [10].

The EE was fractionated under hot reflux and concentrated in a rotary evaporator. This process resulted in four fractions: hexane fraction (FrHex), dichloromethane fraction (FrDcm), ethyl acetate fraction (FrAcOET), and methanolic fraction (FrMeOH) [46]. The presence of quinolinic compounds was monitored by thin-layer chromatography, using silica gel as the stationary phase and hexane and ethyl acetate 4:1 as the mobile phase. The plates were then visualized under visible and UV light. FrDcm presented a greater quantity of quinolinic compounds and was more selective for them, and this was the fraction chosen to be re-fractionated by an open chromatographic column, using silica gel as the stationary phase and solvent gradients with increasing polarity as the mobile phase [10].

The chromatographic column was monitored by thin-layer chromatography (TLC), and fractions with similar profiles were pooled. The sub-fractions that showed precipitates were recrystallized using methanol as solvent. The TLC that presented only one band was taken to NMR, and it was possible to identify the isolation of eleutherin and isoeleutherin. These substances were analyzed by Hydrogen Nuclear Magnetic Resonance (1H NMR) for identification purposes. The ^1^H NMR analyses were performed on a Bruker Ascend 400 spectrometer (operating at 400 MHz for hydrogen). Samples were solubilized in deuterated chloroform (CDCl3). Chemical shifts (δ) were measured in ppm and coupling constants (J) in Hertz (Hz). Tetramethylsilane (TMS) was used as an internal reference [10].

Eleutherin—^1^H NMR 400 MHz (CDCl3): d 1.36 (3H, d, J = 8.0 Hz, Me-3), d 1.53 (3H, d, J = 8.0 Hz, Me-1), d 2.19 (1H, dq, J = 4.0; 16.0 Hz, H4-ax), d 2.74 (1H, dt, J = 4.0; 16.0 Hz, H-4 eq), d 3.58 (1H, m, H-3), d 3.99 (3H, s, OMe9), d 4.85 (1H, m, H-1), d 7.27 (1H, d, J = 8.0 Hz, H-6), d 7.63 (1H, t, J = 8.0; 16 Hz, H-7), and d 7.72 (1H, d, J = 8.0 Hz, H-8); 13C NMR 100 MHz (CDCl3): d 20.92 (Me-3), d 21.38 (Me-1), d 30.05 (C-4), d 56.59 (C-9), d 68.70 (C-3), d 70.40 (C-1), d 117.93 (C-8), d 119.12 (C-7), d 134.66 (C-6), d 120.46 (C-4a), 134.15 (C-11a), d 140.08 (C-5a), d 148.83 (C-9a), d 159.54 (C-9), d 183.84 (C-5), and d 184.15 (C-11) (Figure S6).

Isoeleutherin—^1^H NMR 400 MHz (CDCl3): d 1.32 (3H, d, J = 8.0 Hz, Me-3), d 1.52 (3H, d, J = 8.0 Hz, Me-1), d 2.22 (1H, dq, J = 4.0; 16.0 Hz, H4-ax), d 2.68 (1H, dd, J = 4.0; 16.0 Hz, H-4 eq), d 3.99 (3H, s, OMe-9), d 4.99 (1H, m, H1), d 7.27 (1H, d, J = 8.0 Hz, H-6), d 7.63 (1H, t, J = 8.0; 16 Hz, H-7), and d 7.72 (1H, d, J = 8.0 Hz, H-8); 13C NMR 100 MHz (CDCl3): d 19.93 (Me-3), d 21.67 (Me-1), d 29.98C-4), d 56.62 (OMe-10), d 62.64 (C-3), d 67.58 (C-1), d 117.99 (C-8), d 119.28 (C-7), d 119.93 (C-4a), d 134.26 (C11a), d 134.87 (C-11a), d 139.54 (C-5a), d 148.23 (C-9a), d 159.90 (C-9), d 182.90 (C-5), and d 184.42 (C-10) (Figure S7).

### 4.2. Antimicrobial Activity

The antibacterial activity in vitro was evaluated in accordance with the established standard methodology M7-A6, as outlined by the Clinical And Laboratory Standards Institute—CLSI, 2003 [47], against strains of *Staphylococcus aureus* ATCC 6538 (Gram-positive) and *Escherichia coli* ATCC 32213 (Gram-negative), both obtained from the Central Laboratory of the State of Pará (LACEN). Initially, bacteria were cultured for 24 h (before testing) on nutrient agar at 37 °C in accordance with the recommendations of document M07-A9 ‘Methods for Dilution Antimicrobial Susceptibility Testing for Bacteria That Grow Aerobically’ (incubation temperature of 35 °C ± 2 °C for microdilution testing) [48]. Subsequently, the microbial suspension (inoculum) was prepared; approximately 3 to 4 isolated colonies were transferred to a 4 mL tube with Müller Hinton broth and homogenized in a vortex mixer. The inoculum density was measured using a spectrophotometer, and if it was not between 0.08 and 0.1 (equivalent to an approximate McFarland value of 0.5), adjustments were made by adding colonies. The final inoculum concentration was 1.0 × 10^6^ CFU/mL.

The plates were pre-dosed with 10 µL of each sample solubilized in methanol at concentrations of 1000, 500, 250, 125, 62.5, and 31.25 µg/mL. After solvent evaporation (pre-dosing), 180 µL of Müller Hinton broth and 10 µL of the inoculum were added for a final volume of 200 µL/mL in each. After incubation (35 °C/24 h), 10 µL of triphenyl tetrazolium chloride (TTC) was added to all wells, and the plates were incubated again for 4 h.

Subsequently, the plates were visually read to determine the minimum inhibitory concentration (MIC), defined as the lowest concentration without changing the color of the medium. Then, the absorbances were quantified by a spectrophotometer reader (590 nm) to determine the concentration that inhibits 50% of microbial growth (IC_50_). The IC_50_ determination was performed by linear regression (GraphPad Prism 7.0 program) and expressed as active (IC_50_ < 100 µg/mL), moderately active (IC_50_ between 100 and 500 µg/mL), weakly active (IC_50_ between 500 and 1000 µg/mL), and inactive (IC_50_ greater than 1000 µg/mL) [25].

To ensure the accuracy of the results, the direct effect of the solvent (methanol) on the growth of the strains tested was examined, and all experiments were performed in triplicate.

To determine the minimum bactericidal concentration (MBC), 10 µL of the concentration equal to or greater than the MIC was removed from the well, seeded in Petri dishes containing Müller Hinton agar, and incubated at 35 °C for 24 h. The lowest concentration without bacterial growth was considered the MBC.

### 4.3. Target Proteins Involved in Eleutherin and Isoeleutherin Activity

Eleutherin and isoeleutherin were designed using Marvin Js program. Available online: https://chemaxon.com/products/marvin-js (accessed on 12 October 2023). Prepared for docking in BIOVIA Discovery Studio ^®^ (Dassault Systèmes, version 4.5). The online tool PharmMapper (PharmMapper 2017, available at: http://www.pharmmapper.com, accessed on 20 October 2023) identified potential drug targets of these compounds through reverse pharmacophore matching, combining the compound with an internal database of pharmacophore models [49]. Then, the targets with the highest scores were selected for molecular docking through score adjustment to evaluate and classify the potential interactions between the molecules of interest and several target proteins, based on their three-dimensional conformation involving shape compatibility, binding energy, chemical interactions, and experimental data (crystallography and bioassay data).

The targets were obtained from the Protein Data Bank (PBD) available online: https://www.rcsb.org/ (accessed on 20 October 2023). Subsequently, the interactions between eleutherin, isoeleutherin, and *S. aureus* proteins were explored through molecular docking, based on the preliminary MIC and CBM results.

### 4.4. Molecular Docking

Molecular docking simulations were performed with the naphthoquinones eleutherin and isoeleutherin selected in Marvin Js program. This study used GOLD 2020.1 program [50] from the Cambridge Crystallographic Data Center—CCDC, located in Cambridge, United Kingdom. This software employs a genetic algorithm to explore and select conformations of flexible compounds capable of binding to the active site of a protein [50].

The conformations were evaluated using the GoldScore scoring function with a 100% effective search. To improve the prediction accuracy, ChemScore and GoldScore were selected for this study. All ligand–receptor interactions were analyzed using BIOVIA^®^ software version 4.5.

In GoldScore, affinity is evaluated by physical and chemical interactions between the atoms of the ligand and the protein to calculate the score that represents the stability of this complex. The components that form this field are protein–ligand hydrogen bond energy, external Van der Waals energy, internal Van der Waals energy of the ligand, and the intramolecular hydrogen bond energy of the ligand [51]. The ChemScore function is empirically derived from experimental data of known protein–ligand complexes, allowing parameter adjustments to provide accurate estimates of the binding free energy and, consequently, the affinity of that binding, estimating the total change in free energy that occurs in the binding, using as criteria the hydrogen interactions, hydrophobic interaction area, unfavorable interactions, and binding free energy [49,52].

The higher the GoldScore value, the greater the protein residues’ binding capacities, indicating greater affinity between the ligand and the protein and more stability in the complex [53]. In turn, stability is also determined by the binding free energy (ΔG), where a negative ΔG indicates complex formation is thermodynamically favorable and the complex is stable.

In this sense, the best ligand choice is related to the complex stability, ligand specificity for the target, general affinity, and specific context of application [31], for example, inhibiting bacteria.

Molecular docking predictions were validated through redocking, in which the crystallographic ligand was relocated to the protein’s active site, predicting its conformation. Validation was determined considering only structures whose Root Mean Square Deviation (RMSD) was less than 2 angstroms (Å).

## 5. Conclusions

The inhibitory activity of eleutherin and isoeleutherin against *S. aureus* was observed to have an MIC and MBC of 1000 µg/mL for both compounds. The IC_50_ values were determined to be 165 µg/mL for eleutherin and 172.90 µg/mL for isoeleutherin. Their bacteriostatic activity seems to be related to the PDF and MetAP proteins, respectively. In addition, eleutherin interacts with the QacR regulator, which is involved in bacterial resistance, as it regulates the expression of the efflux pump. Inhibition of this regulator may prevent the bacteria from becoming resistant to different classes of drugs.

Another crucial binding target of eleutherin is BlaR1, a sensor system that regulates resistance to β-lactam antibiotics. Inhibition of this process represents a significant strategy for preventing resistance to the β-lactam class. The docking data indicate that this is the primary target of eleutherin action. Further research should concentrate on corroborating these in silico findings through in vitro and in vivo studies as well as investigating the potential of eleutherin as an adjuvant to enhance the efficacy of β-lactam antibiotics. Furthermore, additional studies should be conducted to evaluate its impact on other resistant strains and its broader implications in combating antimicrobial resistance.

## Figures and Tables

**Table 1:** *Staphylococcus aureus* and *Escherichia coli* inhibition at different concentrations of eleutherin and isoeleutherin.

Concentration (µg/mL)	*Staphylococcus aureus*		*Escherichia coli*	
	Inhibition (%)			
	Eleutherin	Isoeleutherin	Eleutherin	Isoeleutherin
1000	99.7	99.4	30.2	30.0
500	94.0	94.4	30.0	30.2
250	59.5	52.5	30.3	29.6
125	47.7	48.7	27.0	27.4
62.5	35.6	28.4	25.8	25.2
31.2	12.5	7.8	23.1	22.8

**Table 2:** Antibacterial activity of eleutherin, isoeleutherin, and chloramphenicol against *Staphylococcus aureus* and *Escherichia coli*.

Compounds	*Staphylococcus aureus*			*Escherichia coli*		
	MIC (µg/mL)	IC_50_ (µg/mL)	MBC (µg/mL)	MIC (µg/mL)	IC_50_ (µg/mL)	MBC (µg/mL)
Eleutherin	1000	165.00	1000	>1000	>1000	>1000
Isoeleutherin	1000	172.90	1000	>1000	>1000	>1000
Chloramphenicol	250	78.2	-	125	71.33	-

IC_50_—Concentration that inhibits 50% of microbial growth; MIC—minimum inhibitory concentration; MBC—minimum bactericidal concentration; active (IC_50_ < 100 µg/mL), moderately active (IC_50_ between 100 and 500 µg/mL), weakly active (IC_50_ between 500 and 1000 µg/mL), and inactive (IC_50_ greater than 1000 µg/mL); and chloramphenicol—positive control.

**Table 3:** Targets of eleutherin and isoeleutherin. PDB—Protein Data Bank.

	PDB Code	Target Name	Score Adjustment
Eleutherin	1Q1Y	Peptide deformylase (PDF)	1.56
	1RKW	Regulator QacR	1.37
	1XA7	Regulatory protein BlaR1	1.07
Isoeleutherin	1QXY	Methionine aminopeptidase (MetAP)	2.48

**Table 4:** Docking parameter of *S. aureus* target proteins with naphthoquinones and crystallographic ligands.

*Staphylococcus aureus* Target	Compound	CS	ΔG	GS	WdWExt
Peptide deformylase (PDF)	Eleutherin	23.00	−25.47	28.58	20.62
	Actinonin *	28.45	−29.46	62.07	41.12
Regulator QacR	Eleutherin	33.79	−33.86	29.53	21.30
	Pentamidine *	36.58	−36.62	56.31	39.71
Regulatory protein BlaR1	Eleutherin	28.67	−28.71	35.19	21.68
	Benzylpenicillin *	27.01	−27.39	35.01	22.72
Methionine aminopeptidase (MetAP)	Isoeleutherin	21.79	−22.33	33.72	33.72
	Ketoheterocycle 618 *	23.00	−24.49	45.79	29.97

CS—ChemScore; GS—GoldScore; WdWExt—Van der Waals interactions; ΔG—energy variation; and * reference ligand used for comparison.

## Data Availability

Data are not contained within the article and supplementary materials.

## Supplementary Materials

The following supporting information can be downloaded at https://www.mdpi.com/article/10.3390/ijms252312583/s1.
