# Supplementary material for: Eleutherin and Isoeleutherin Activity against Staphylococcus aureus and Escherichia coli Strain’s: Molecular Docking and Antibacterial Evaluation
Source: Int J Mol Sci. 2024 Nov 23;25(23):12583. doi: 10.3390/ijms252312583 (PMC11641138; doi:10.3390/ijms252312583)
Supplement: Supplementary file 1 [file ijms-25-12583-s001.zip › ijms-3196318-supplementary.pdf]

Supplementary Material

Figure S1. Chemical constituents isolated from *Eleutherine plicata*

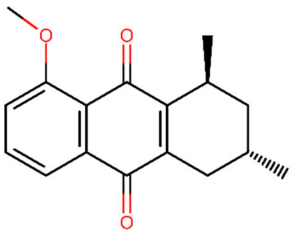

(A)

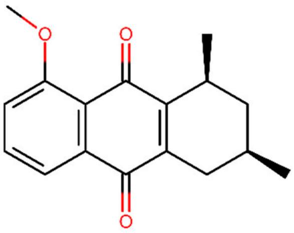

(B)

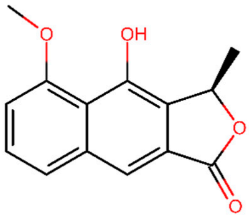

(C)

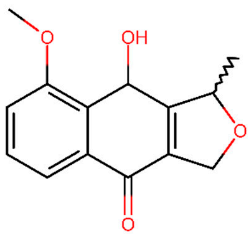

(D)

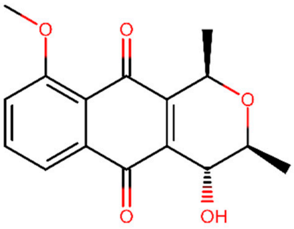

(E)

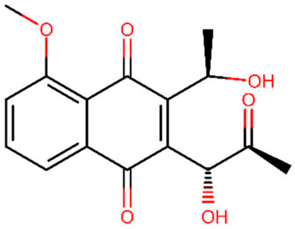

(F)

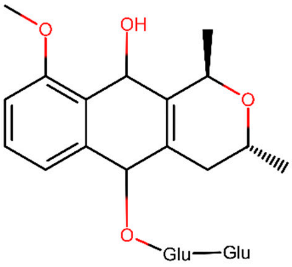

(G)

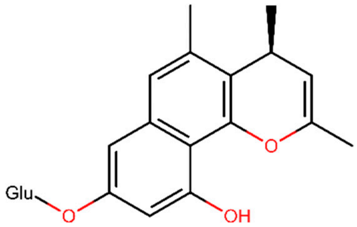

(H)

(**A**) isoeleutherin; (**B**) eleutherin; (**C**) eleutherol; (**D**) eleutherinone; (**E**) (R) −4-Hydroxyeleutherin; (**F**) eleutherone; (**G**) isoeleuthoside C; (**H**) eleutherinol-8-O-β-D-glucoside.

Figure S2. Microdilution Assay of Eleutherin and Isoeleutherin Samples Against *Staphylococcus aureus*

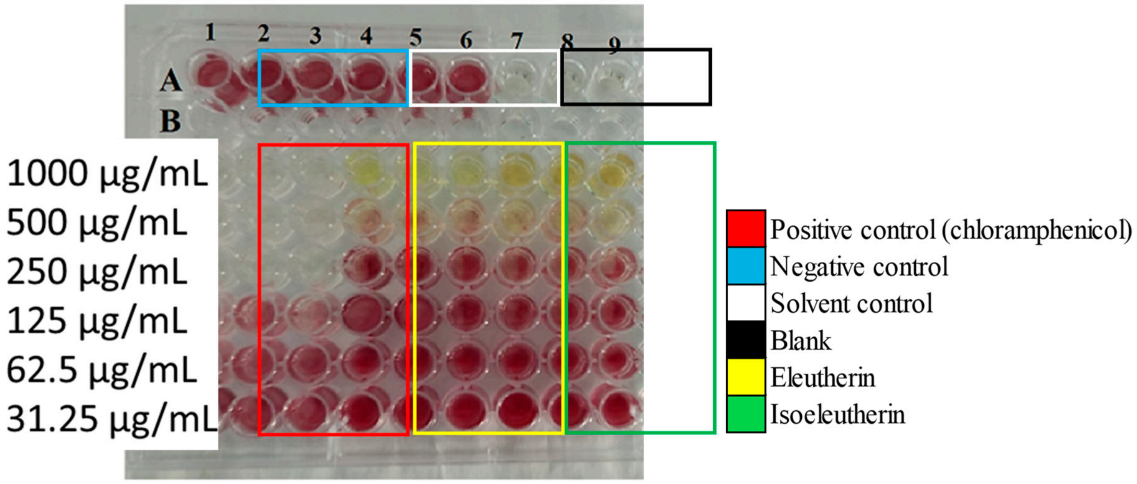

Figure S3. Microdilution Assay of Eleutherin and Isoeleutherin Samples Against *Echerichia coli*

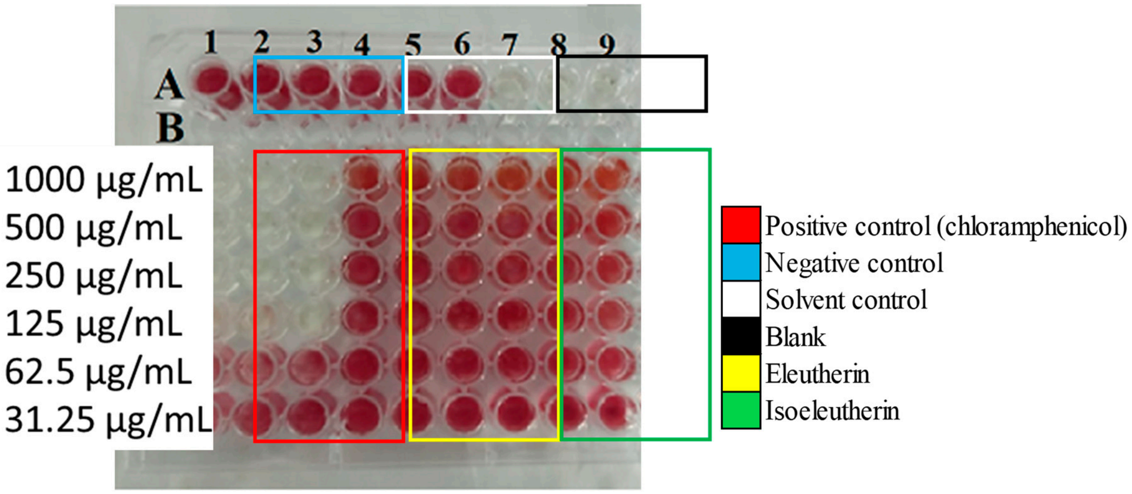

Figure S4. MBC of *Staphylococcus aureus* on Müller Hinton agar

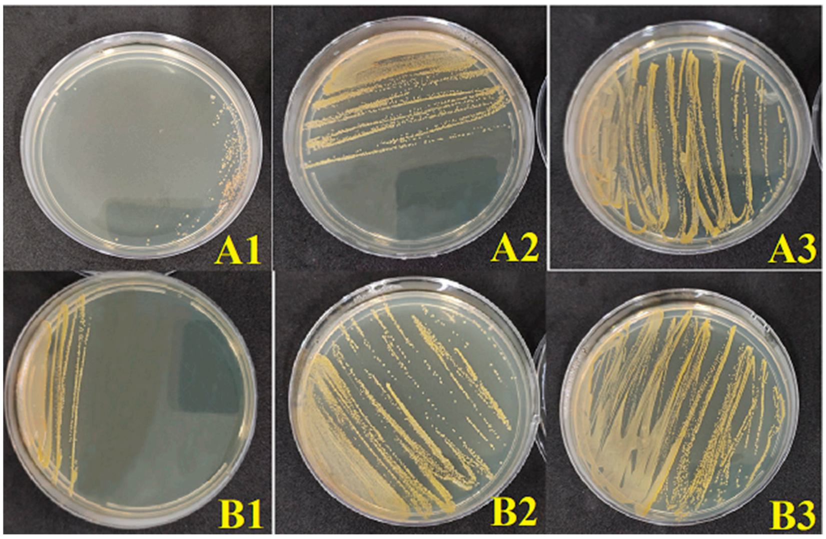

Subtitle: A1 - Eleutherin 1000 µg/mL; A2 - Eleutherin 500 µg/mL; A3 - Eleutherin 250 µg/mL; B1 Isoeleutherin 1000 µg/mL; B2 - Isoeleutherin 500 µg/mL; B3 - Isoeleutherin 250 µg/mL

Figure S5. MBC of *Escherichia coli* on Müller Hinton agar

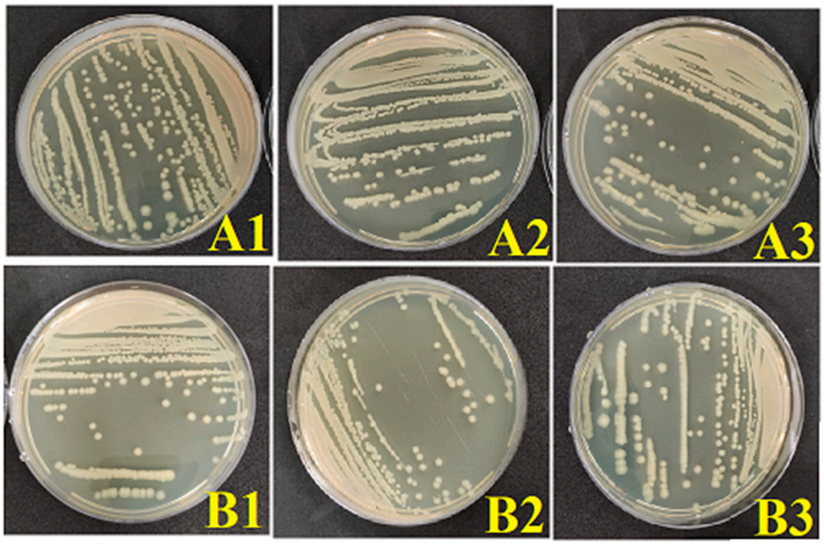

Subtitle: A1 **-** Eleutherin 1000 µg/mL; A2 - Eleutherin 500 µg/mL; A3 - Eleutherin 250 µg/mL; B1 Isoeleutherin 1000 µg/mL; B2 - Isoeleutherin 500 µg/mL; B3 - Isoeleutherin 250 µg/mL

Figure S6. ^{1}H spectrum of Fr 22-23. Conditions: deuterated chloroform solvent and TMS as internal calibration, spectrum obtained on a 400 MHz device.

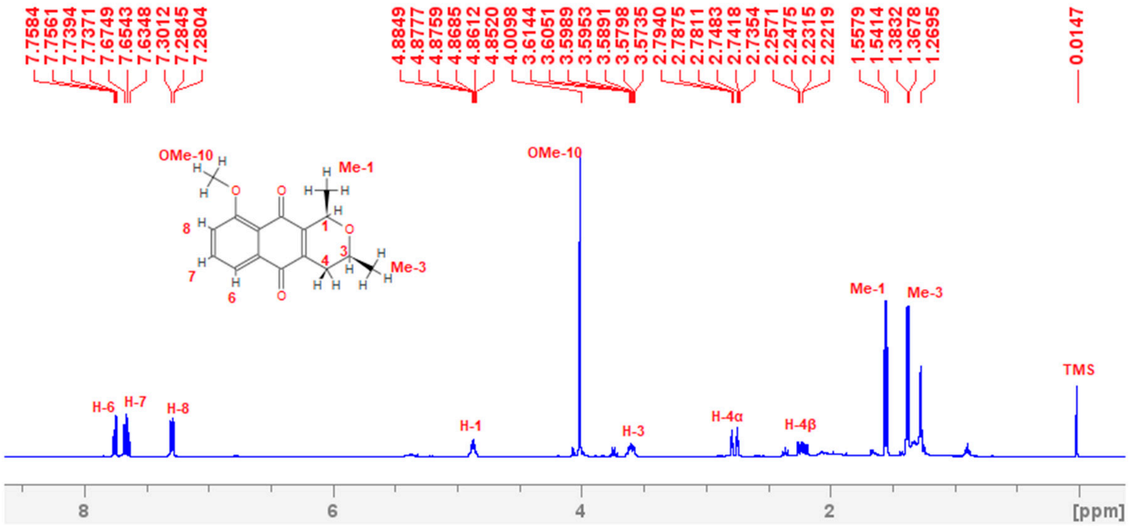

Figure S7. ^{1}H spectrum of Fr 27. Conditions: deuterated chloroform and TMS as internal calibration, spectrum obtained on a 400 MHz device

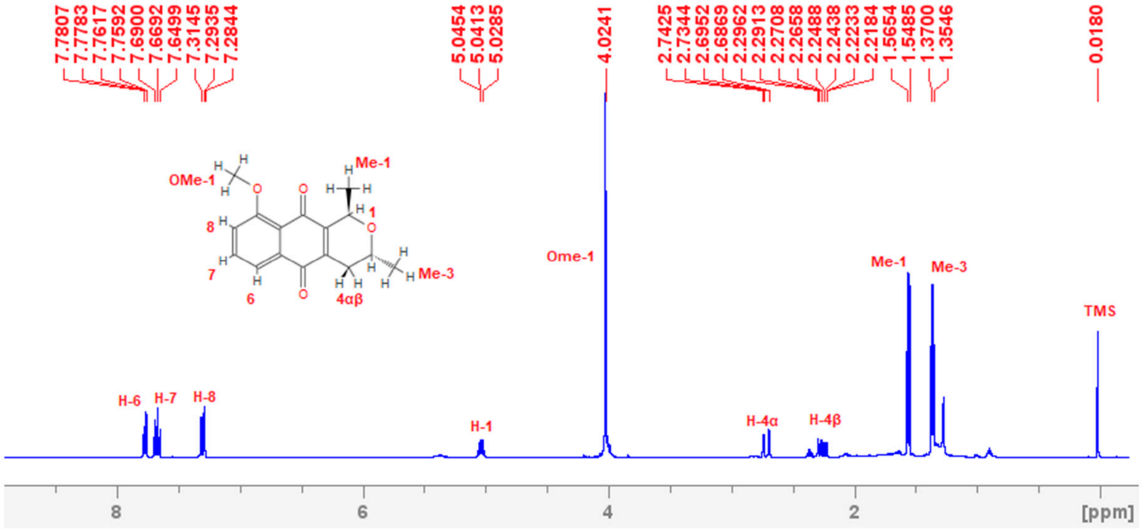

Table S1. Interactions of eleutherin, isoeleutherin and reference compounds with *Staphylococcus aureus* targets

**Peptide deformylase** – PDF				
	**Eleutherin**		**Actinonin**	
Amino acid	Bond	Distance (Å)	Bond	Distance (Å)
VAL 59	Alkyl	4.65	Alkyl	4.15
VAL 59	Pi-alkyl	5.81	-------	----
VAL 151	Pi-alkyl	6.30	Alkyl	4.16
GLU 155	Unfavorable bump	2.99	Van der Waals	----
HIS 154	Alkyl	5.35	Unfavorable Donor-Donor	4.04
HIS 154	-------		Pi- sigma	3.67
HIS 154	-------		Alkyl	4.52
LEU 112	Alkyl	4.48	Van der Waals	----
LEU 112	Alkyl	4.28	Van der Waals	----
ARG 56	Alkyl	3.87	Van der Waals	----
**Transcriptional regulator QacR**				
	**Eleutherin**		**Pentamidine**	
TYR 93	Pi-pi – stacked	5.29	Pi-sigma	3.16
TYR 93	Alkyl	4.89	-------	----
TYR 123	Pi- Alkyl	6.63	Alkyl	5.07
TYR 123	Alkyl	6.02	-------	----
LEU 119	Alkyl	5.63	-------	----
PHE 162	Alkyl	5.38	-------	---
LEU 54	Alkyl	4.80	-------	----
ILE 99	Alkyl	5.44	-------	----
TYR 103	Alkyl	4.83	-------	
LYS 60	-------	----	Alkyl	5.24
ALA 153	-------	----	Conventional Hydrogen bond	3.56
**Regulatory protein BlaR1**				
	**Eleutherin**		**Benzylpenicillin**	
TYR 199	Pi- sigma	3.86	Van der Walls	----
ILE 201	Alkyl	5.30	Van der Walls	----
TYR 206	Alkyl	5.57	Alkyl	6.22
TYR 206	-------	---	Alkyl	5.32
PHE 91	Van der Waals	----	Pi- Sulfur	6.83
PHE 91	Van der Waals	----	Alkyl	6.84
**Methionine aminopeptidase - MetAp**				
	**Isoeleutherin**		**Ketoheterocyclo 618**	
HIS 76	Pi -pi T- shaped	5.62	Van der Waals	----
HIS 76	Pi -pi T- shaped	5.83	Van der Waals	----
HIS 76	Alkyl	5.56	Van der Waals	---
HIS 175	Pi -pi T- shaped	6.73	Pi- sulfur	7.12
HIS 175	Pi -pi T- shaped	6.01	-------	---
HIS 175	Alkyl	5.66	-------	---
LEU 174	Alkyl	5.83	Alkyl	5.00
LEU 174	Pi- alkyl	5.66	-------	---
PHE 204	Alkyl	6.46	Van der Waals	---
